# Effects of Hemoglobin-Based Oxygen Carriers on Blood Coagulation

**DOI:** 10.3390/jfb5040288

**Published:** 2014-12-12

**Authors:** Kimia Roghani, Randall J. Holtby, Jonathan S. Jahr

**Affiliations:** 1 Department of Anesthesiology and Perioperative Medicine, David Geffen School of Medicine at UCLA, Ronald Reagan UCLA Medical Center, 757 Westwood Plaza, Suite 3325, Los Angeles, CA 90095, USA; 2 Department of Biology, College of Arts and Sciences, Syracuse University, Syracuse, NY 13244, USA; E-Mail: kroghani@syr.edu; 3 School of Medicine, St. George’s University, Grenada, West Indies; E-Mail: rholtby@sgu.edu

**Keywords:** HBOC, blood substitute, resuscitation, bleeding, coagulopathy

## Abstract

For many decades, Hemoglobin-based oxygen carriers (HBOCs) have been central in the development of resuscitation agents that might provide oxygen delivery in addition to simple volume expansion. Since 80% of the world population lives in areas where fresh blood products are not available, the application of these new solutions may prove to be highly beneficial (Kim and Greenburg 2006). Many improvements have been made to earlier generation HBOCs, but various concerns still remain, including coagulopathy, nitric oxide scavenging, platelet interference and decreased calcium concentration secondary to volume expansion (Jahr *et al.* 2013). This review will summarize the current challenges faced in developing HBOCs that may be used clinically, in order to guide future research efforts in the field.

## 1. Introduction

Considering that a staggering 80% of the world population does not have access to fresh blood products, the development of HBOCs is imperative [1]. However, before HBOCs may be administered, some issues such as coagulopathy, nitric oxide scavenging, platelet interference and decreased calcium concentration secondary to volume expansion must be addressed [2]. More than 40,000 units of blood are transfused daily, resulting in more than 15 million units of blood transfused annually [3]. Although usually of benefit, blood transfusions have major risks of complications and high costs. The availability of blood is limited as a donated product, as is its applicability and shelf life. The availability of blood is even more reduced in remote areas and combat zones [1]. Blood for transfusions must also be refrigerated and a high volume is needed. The costs as well are high, estimated at over $1000/unit [4]. If there is enough blood available to transfuse, there are numerous possible complications related to the transfusion. These include transmitted disease (malaria, Chagas disease, HIV, Hepatitis C, *etc.*), wound infection, compatibility issues, immunomodulation, transfusion related acute lung injury (TRALI), and transfusion associated circulatory overload (TACO). Transfusion associated immunomodulation (TRIM) and its effects include increased prevalence of cancer recurrence and postoperative bacterial wound infections, indicating an unmet need for blood substitutes [5]. TRALI has an incidence between 0.08%–1.5% and, a mortality rate of 0.4%–1.5% per unit transfused [6]. This review will cover the assessment of coagulation, the *ex vivo*, translational, and *in vivo* models, the potential mechanisms of coagulopathy, and the effects of Hemoglobin-based Oxygen carriers on platelet function.

## 2. History

The earliest generations of the Hemoglobin-based Oxygen carriers (HBOC) had significant complications. The early generations had a high affinity for oxygen due to the loss of 2,3 DPG during purification [1]. Also, nitric oxide scavenging occurred, which caused vasoconstriction and hypertension [7]. The early generations also demonstrated short retention time [1]. The later generations implemented polymerization and cross-linking, which decreased vasoconstriction. These generations also had a decreased oxygen affinity. The most recent HBOCs are low hemoglobin, high affinity, and in some cases increased viscosity solutions, however these strategies for oxygen delivery and resuscitation are still being vetted.

## 3. Coagulation Assessment

Measuring the efficacy of these products is often difficult because most of the clinical laboratory equipment’s original use was not intended for evaluating HBOCs, which may lead to erroneous results. Therefore, choosing the appropriate analyzer for testing is key. The two main methods used to detect coagulation are optical and mechanical clot detection. However, there is still an issue concerning the validation of these methods. Plasma hemoglobin may interfere with optical testing and although mechanical instruments have proven to be less susceptible to interference, issues still arise. It is for this reason that all instruments must be verified in order to insure that correct clinical interpretations can be drawn. The entire process of blood coagulation has been monitored using Thromboelastography (TEG) and Thromboelastometry (ROTEM) [8,9]. In place of a plasma sample, TEG and ROTEM use whole blood samples, which enables them to assess the qualitative state of the hemostasis process that depends on quantity as well as functional status of platelets, plasma clotting factors (for complete list of clotting factors refer to Table 1) and fibrinolytic system [2]. TEG assesses platelet function by utilizing different anticoagulants and differential platelet (PLT) activators. Another method of assessing platelet function is the PFA-100. The PFA-100 imitates high shear stress *in vivo* conditions by using a capillary apparatus to measure platelet function [2]. This method is not only faster but also more sensitive in recognizing platelet function defects [10]. While assessing platelet function during coagulation, the coagulation cascade must also be considered.

**Table 1:** Clotting factors: name, description, function [11].

Name	Description	Function
Fibrinogen (Factor I)	MW = 340,000 Da; glycoprotein	Adhesive protein that forms the fibrin clot
Prothrombin (Factor II)	MW =72,000 Da; vitamin K-dependent serine protease	Activated form is main enzyme of coagulation
Tissue factor (Factor III)	MW = 37,000 Da; also known as thromboplastin	Lipoprotein initiator of extrinsic pathway
Calcium ions (Factor IV)	Necessity of Ca^++^ ions for coagulation reactions described in 19th century	Metal cation necessary for coagulation reactions
Labile factor (Factor V)	MW =330,000 Da	Cofactor for activation of prothrombin to thrombin
Proconvertin (Factor VII)	MW = 50,000 Da; vitamin K-dependent serine protease	With tissue factor, initiates extrinsic pathway
Antihemophilic factor (Factor VIII)	MW = 330,000 Da	Cofactor for intrinsic activation of factor X
Christmas factor (Factor IX)	MW = 55,000 Da; vitamin K-dependent serine protease	Activated form is enzyme for intrinsic activation of factor X
Stuart-prower factor (Factor X)	MW = 58,900 Da; vitamin K-dependent serine protease	Activated form is enzyme for final common pathway activation of prothrombin
Plasma thromboplastin antecedent (Factor X)	MW = 160,000 Da; serine protease	Activated form is intrinsic activator of factor IX
Hageman factor (Factor XII)	MW = 80,000 Da; serine protease	Factor that normally starts aPTT-based intrinsic pathway
Fibrin stabilizing factor (Factor XIII)	MW =320,000 Da	Transamidase that cross-links fibrin clot
High molecular weight kininogen (Fitzgerald, Flaujeac, or William factor)	MW = 110,000 Da; circulates in a complex with factor XI	Cofactor
Prekallikrein (Fletcher factor)	MW = 85,000; serine protease	Activated form that participates at beginning of aPTT-based intrinsic pathway

## 4. Models: *Ex Vivo*, Translational, and *in Vivo*

In the *ex vivo* model, the larger zero linked hemoglobin polymer (OxyVita^®^, OXYVITA, Inc., New Windsor, NY, USA) was similar to the Hemoglobin glutamer (bovine), (Oxyglobin^®^, HBOC-200, HbO_2_ Therapeutics LLC, Philadelphia, PA, USA) in terms of coagulopathy, despite the difference in molecular weight. This result suggests that greater coagulopathy is not inherent with extensive polymerization in HBOC products [2]. However, at high and very high dilutions, Zero-linked Hb polymer and HBOC-200 products exhibited a decrease in clot tensile strength by 33% and 49%, respectively, compared with 6% hetastarch. While the mechanism for this difference has not been elucidated, use of Zero-linked Hb polymer at the small volumes recommended by the manufacturer (2–3 mL/kg), which corresponds to a dilution less than the 1:11 “low dilution”, should not increase risk of clinical bleeding. In the translational model, resuscitation with HBOC-201 did produce a mild dilutional coagulopathy, similar to resuscitation with 6% hetastarch (HEX) when compared to no resuscitation [12]. In the *in vivo* human study, Hemoglobin gluatmer (bovine) 201 (HBOC-201, Hemopure) was tested against packed red blood cell (PRBC). There was mild platelet dysfunction [13].

## 5. Coagulopathy: Potential Mechanisms

Potential mechanisms for coagulopathy with HBOCs include dilutional coagulopathy/hypocalcemia, oxidation to methemoglobin inhibiting platelet aggregation, large molecular weight molecules complexing with von Willebrand factor and speeding its elimination, and Nitric oxide scavenging. Many studies have imitated hemodilution during clinical resuscitation of hemorrhagic shock with increasing doses of HBOCs compared to crystalloid or colloid fluids, such as hetastarch. These results indicate a shared dilutional coagulopathy among the previously mentioned products. Crystalloid, colloid, and Zero-link Hb polymer had a tendency to shorten *R* at lower dilutions then slowly lengthen with higher dilutions. The measurement *R* is defined as the latency period between placement of blood in the TEG analyzer and start of clot formation. This initial hypercoagulable effect is thought to be propagated by a greater sensitivity of anticoagulants such as antithrombin III to the effects of mild dilution compared with coagulation factors, thrombin, and other factors in the coagulation cascade (Refer to Table 1 for all clotting factors). There was an increase in coagulability for colloids and crystalloids at up to a 40% dilution and 50% dilution, respectively. The threshold was discovered to be 70% dilution due to evidence of hypocoagulation [14].

*In vitro* dilution with normal saline, polyhemoglobin solution, 5% bovine albumin at 25% and 50% dilution did not change TEG parameters *R* (reaction time; time to clot initiation) and *K* (rate of clot development), significantly [15]. Stroma-free hemoglobin did show a moderate procoagulant trend at 25% and 50% dilution with shortened *R* and *K* times. A possible mechanism for this is that stroma-free hemoglobin might be less stable in solution, causing auto-oxidation of hemoglobin to methemoglobin, thereby releasing superoxide radicals that may initiate procoagulant processes such as cell damage and platelet activation. There was a decrease in the clot strength parameter MA (maximum amplitude) within all groups with increasing dilution, which points to a mechanism of dilution. Dilutional explanation for this variable is a consistent interpretation since clot strength does seem to be correlated with hematocrit and platelet counts [15].

Although there was no difference in platelet count or fibrinogen concentration between low and medium weight hydroxyethyl starches, there was a decrease in factor VIII and von Willebrand factor (vWF) activity that was more noticeable in the medium weight fluid (HES200) compared to the low weight fluid (HES70) [16]. There was also greater increase in aPTT (activated partial thromboplastin time) and a more significant decrease in MA and α (rate of clot development) by HES200 compared to HES70. Considering both TEG parameters are influenced by platelet count and function, and taking into account that there was no difference in platelet count between the HES solutions, the difference may be due to the more pronounced decrease in vWF and its role in platelet linking. The most probable mechanism for the decrease in vWF is enhanced elimination. It is suggested that larger HES molecules form complexes with the vWF to be cleared from the circulation. Similarly, the considerable increase in aPTT may be an indication of the greater decrease in vWF and factor VIII by the larger molecular weight fluid. A pressing concern is that similar mechanisms may be present with HBOCs, since HBOCs are also large molecular weight compounds.

HBOC-201, showed no significant difference in TEG parameters at clinically relevant concentrations during a trial in South Africa for the treatment of adult surgical patients. It was tested *in vitro* against lactated Ringer’s (LR) solution [17]. Whole blood was diluted with HBOC-201 and LR, and at concentration of 2 g/dL, both fluids showed statistically significant shortened *R* and *K* times with an increased α angle compared to undiluted control samples. These results suggest a possible procoagulant effect at a HBOC-201 concentration of 2 g/dL, which correlates to about 20% hemodilution. In contrast to the above studies, MA did not vary significantly from undiluted control in any of the tested samples. However, the only statistically significant difference between HBOC-201 and LR in this study was a slight decrease in MA by HBOC-201 compared to LR, which marginally increased MA. MA reflects the properties of platelets, fibrinogen and factor XIII in contributing to clot strength, so this may reflect a minor effect on platelet function by HBOC-201. Similarly, a swine model of hemorrhagic shock, suggests hemodilution of vWF plays a role in the coagulopathic effects of hemodilution with HBOC-201 [18].

Nitric oxide scavenging has been an issue with HBOCs. In the human study however, there was no apparent effect on human platelet activation or function [19]. There could be a possible increase in hemostasis due to nitric oxide scavenging by free hemoglobin [20]. In a study comparing hemoglobin raffimer and albumin in rabbits there was evidence of vasoconstriction and aggregation of stimulated platelets (increased activation) [20]. Overall, it appears that clinically tested HBOCs do not produce coagulopathy at clinically relevant doses, however there is a need for a greater number of subjects and post-marketing data [21,22].

## 6. HBOC Effects on Platelet Function

Platelet function is an important determinant of coagulation. Coagulopathy has been shown to correlate with the content of high molecular weight polymers present in hetastarch solutions [23]. Studies have proposed that the hetastarch binds to the coagulation factors and the surfaces of the red blood cells and platelets, causing an accelerated clearance of coagulation factors and a decrease in platelet activation [24,25,26].

The oxidation to methemoglobin inhibits platelet aggregation. In studies, there was an impairment in clot propagation and strength in the high methemoglobin samples. There was evidence of platelet modification of redox sensitive sites involved in platelet aggregation and activation [27]. In an *ex vivo* study, previously opened packages of HBOC-200 reached 65% methemoglobin concentration compared to a 1% methemoglobin concentration in freshly opened bags [27]. Measuring TEG parameters showed statistically significant impairment in clot propagation and strength in the high methemoglobin samples. However, this is an issue that can be easily fixed if the accumulation of methemoglobin is prevented.

## 7. Discussion

Dilutional coagulopathy is to be expected from a large fluid resuscitation with any fluid devoid of clotting factors or platelets, and so is not limited to HBOCs [12,13,21,28]. Studies have shown no increase in coagulopathy with HBOCs when compared to hetastarch fluids [2]. If fresh samples are used, methemoglobin may not be clinically relevant [27]. Also, at clinically relevant doses, HBOCs do not cause more platelet dysfunction compared to other resuscitative fluids. The extent of the vasoconstriction and hypertension decreases as the size of the HBOC increases [29]. There is some evidence that shows that p50 is better, although there is no definite proof, there is some evidence. HBOCs with high p50 can release excessive amounts of oxygen into the systemic circulation, which induces vasoconstriction [20]. In regard to vasoconstriction due to nitric oxide scavenging, the major implications are still being debated but as previously mentioned, there is no apparent effect on human platelet activation or function [30]. A detailed study on preventing vasoconstrictive activity can be read in *Oxygen Carriers* (“*Blood Substitutes*”). In addition, a complete list of strategies created to limit hemoglobin binding to nitric oxide can be seen in *Oxygen Therapeutics: Can We Tame Haemoglobin* [31]. Then there comes the issue of the size balance. While polymerization of the hemoglobin causes bigger HBOCs with low p50 might be a more effective resolution for the prevention of vasoconstriction and lead to suitable blood substitutes, large HBOCs can also increase the likelihood of coagulopathy [32,33]. There is some recent evidence [34], that *in vitro* and maybe *in vivo* that certain fish, that live in cold conditions may have hemoglobin polymerization and cell sickling. This phenomenon supports the hypothesis that the response may be due to stressful environmental conditions. However, the relevance or applicability of these findings is not clear as it pertains to models for development of HBOCs or coagulation relationships between structure and function of HBOCs.

## 8. Summary

While polymerization of hemoglobin creates larger HBOCs, and with low p50 might be a more effective resolution for the prevention of vasoconstriction and lead to suitable blood substitutes; larger HBOCs may also increase the likelihood of coagulopathy.
